# Analysis of mucociliar clearance: a new diagnostic method and a therapeutical proposal

**DOI:** 10.1186/2045-7022-5-S4-P16

**Published:** 2015-06-26

**Authors:** Alberto Macchi, Stefania Gallo, Giulia Montrasio

**Affiliations:** 1 ORL Clinic University Insubria, Varese, Italy; 2 ORL Clinic University of Insubria, ORL Clinic, Varese, Italy

## 

Mucociliary clearance is a key defence mechanism in human upper and lower airways and its impairment predisposes to chronic infections of the nose, paranasal sinuses and respiratory tract. Nasal mucociliary clearance is a first-line physiological function of nasal cavity, that helps protecting the lower respiratory tract from undesirable organic and inorganic matter, including microorganisms. On one hand, multiple investigations have demonstrated a marked decrease in nasal mucociliary clearance in patients with rhinitis or chronic rhinosinusitis. Ciliary motility analysis allows us to have an index of our defense mechanisms functionality and of our ability to react to microorganisms. The ciliary motility can be evaluated with a light microscope using phase contrast. Sampling should be performed through a brushing at the level of the middle third of the inferior turbinate and the sample must be analyzed immediately after collection. Through the analysis of the sample, it is possible to evaluate the presence of ciliated cells with a functional edge, to calculate the length of the ciliary motilià, to assess the direction of movement and to evaluate the coordination of the lashes. The time needed for this examination is short, it only takes 5 to 10 minutes, and it appears to be not invasive, repeatable and not painful. The ciliary function may also be influenced by the use of certain medications, such as anti-histamines, whereas it may be stimulated and improved by the use of topical hyaluronic acid. HA may be an important regulator of the inflammatory process. During nasal inflammation, high-molecular weight HA breaks down under the influence of free radicals and enzymes. Low-molecular weight fragments deliver signals regarding tissue damage and mobilize immune cells, while the high molecular weight form suppresses immune cells function and prevents excessive exacerbation of inflammation. Based on the known background of the effects of HA in the regulation of vasomotor tone, mucous gland secretion and in the modulation of the inflammatory process, it is suggested that HA may improve ciliary motility, nasal mucosa cellularity and inflammatory status.

